# How static and kinetic meditation, with or without guidance, affect autonomic nervous system activity in novice meditators

**DOI:** 10.3389/fpsyg.2025.1572499

**Published:** 2025-08-05

**Authors:** Jinwoo Han, Myungji Lee, Teri Kim

**Affiliations:** ^1^Department of Arts Therapy, Daegu Catholic University, Gyeongsan, Republic of Korea; ^2^Arts Therapy Center, Daegu Catholic University, Daegu, Republic of Korea; ^3^Department of Physical Education, Kyungpook National University, Daegu, Republic of Korea; ^4^Division of Health and Sport Science, Dongguk University-WISE, Gyeongju, Republic of Korea

**Keywords:** static meditation, kinetic meditation, heart rate variability, autonomic nervous system, sympathetic nervous system

## Abstract

**Objective:**

This study explored the autonomic nervous system responses and perceived experiences of novice meditators during kinetic and static meditation.

**Methods:**

Thirty-five participants completed both meditation types in randomized order. Each 20-min session included 10 min of guided and 10 min of unguided meditation. Heart rate (HR) and Heart rate variability (HRV) was recorded using the Polar H10 and EliteHRV apps. A visual analog scale (VAS) assessed focused attention, peace and calm, and drowsiness.

**Results:**

Focused attention was significantly higher in kinetic meditation than in static meditation and was also higher during guided sessions. Static meditation induced greater drowsiness than kinetic meditation, especially in the unguided condition. All meditation conditions increased HR compared to rest, with guided meditation showing a higher HR than unguided meditation. HRV indices reflected increased sympathetic activity during guided meditation sessions, likely due to cognitive effort in maintaining attention and processing instructions.

**Conclusion:**

For meditation-naïve individuals, movement-based meditation with clear guidance may serve as a more accessible entry point. Kinetic meditation appears to facilitate attentional engagement while mitigating drowsiness, and may remain manageable even when self-administered without guidance. These findings provide an empirical basis for optimizing meditation intervention design, particularly for novice practitioners who may initially perceive meditation as inaccessible or impractical.

## 1 Introduction

The global meditation app market has grown substantially, especially since the COVID-19 pandemic, which has increased awareness of mental health challenges. In 2019, the market value for meditation apps was USD 270.39 million, with projections estimating it to reach USD 4.21 billion by 2027 (Polaris Market Research, 2020). Despite this rapid expansion, many meditation apps suffer from a lack of evidence-based content, low-quality offerings, and a limited understanding of their mechanisms. For instance, among the 700 meditation apps on the iTunes Store, only 23 provide genuine meditation training, and only one is supported by empirical evidence (Mani et al., 2015). This lack of scientific validation exposes users to potentially ineffective interventions (Torous and Firth, 2016).

The meditation techniques of these apps focus on static seated meditation, with few incorporating kinetic forms of meditation that involve physical movement. An analysis of 16 popular iPhone meditation apps revealed that all featured static guided meditation, and none included kinetic approaches (Dauden Roquet and Sas, 2018). This gap in meditation content may arise from a limited understanding of the different forms of meditation. Many developers create apps based on assumptions of or imitating successful ones, with effectiveness testing often occurring after development, which can lead to a “digital placebo” effect (Torous and Firth, 2016; Dauden Roquet and Sas, 2018). Additionally, the overall quality of meditation apps is suboptimal, with an average Mobile Application Rating Scale (MARS) score of 3.2 out of 5.0 for 700 meditation apps (Mani et al., 2015). These factors highlight the critical need for a rigorous investigation into the mechanisms and effectiveness of various meditation forms.

The increasing popularity of meditation has driven extensive scientific research across fields, such as psychology, medicine, and neuroscience. Publications on meditation grew from fewer than 100 in 2006 to 2,808 in 2020, with an average annual growth rate of 23.5% between 2010 and 2020 (Baminiwatta and Solangaarachchi, 2021). These studies focused primarily on the physical and psychological benefits of meditation-based interventions. Meditation techniques are broadly categorized into static and kinetic forms. Static meditation includes seated practices such as mindfulness, mantra meditation, and body scans, whereas kinetic meditation includes movement-based practices such as Hatha yoga, walking meditation, dance, qigong, and tai chi. Hybrid approaches such as Mindfulness-Based Stress Reduction (MBSR), combine static and kinetic elements (Hunt et al., 2018).

Despite the documented benefits of meditation, challenges such as publication bias and negative user experiences are often overlooked. A meta-analysis of 39 studies on meditation revealed a tendency to report only positive results (Eberth and Sedlmeier, 2012). In practice, individuals encounter obstacles such as drowsiness, discomfort, boredom, and negative emotions during static meditation (Sparby, 2022). For example, maintaining focus during a 30-minute body scan without falling asleep is difficult, particularly for beginners, children, and those with physical limitations (Kim, 2013). These challenges make static meditation unsustainable.

Research suggests that kinetic meditation offers enhanced benefits over static meditation. A meta-analysis comparing various meditation types found that while static meditation interventions produce medium effect sizes (Cohen's *d* = 0.4–0.5), kinetic forms like yoga have a larger effect size (Cohen's *d* = 0.77) for improving psychological well-being (Rose et al., 2020). Programs such as MBSR, which incorporate yoga, have demonstrated greater efficacy in promoting psychological wellbeing than do static practices alone (Grossman et al., 2004; Eberth and Sedlmeier, 2012). These findings highlight the potential of kinetic elements to enhance the positive effects of meditation, despite limited research on kinetic meditation. Matko and Sedlmeier (2019) who identified 309 meditation techniques, categorizing them into seven clusters, including meditation with movement, pointed out the lack of research on movement-based meditation techniques and highlighted the need for studies on their specificities and working mechanisms, as well as comparisons with other basic meditation techniques.

Heart rate variability (HRV), a physiological marker of health, has garnered attention due to technological advances in its measurement. HRV reflects the time variability between heartbeats and is linked to vagus nerve activity, a key component of the parasympathetic nervous system (PNS). Higher HRV indicates better autonomic nervous system (ANS) regulation and stress recovery, whereas lower HRV is associated with chronic stress and mental health disorders (McCraty and Shaffer, 2015). Meditation practices, particularly mindfulness and focused breathing, improve HRV and provide an objective measure of physiological benefits (Christodoulou et al., 2020). However, the effects of static and kinetic meditation on HRV and ANS regulation have rarely been directly compared.

Studies on attention-focused static meditation have shown changes in HRV metrics, such as increased standard deviation of NN intervals (SDNN), root mean square of the successive differences (RMSSD), and high-frequency (HF) power, along with decreased low-frequency (LF) power and LF/HF ratio, suggesting enhanced PNS activity (Nesvold et al., 2012; Krygier et al., 2013; Arredondo et al., 2017). A meta-analysis of 17 RCTs on Tai Chi and Yoga also found similar trends, including decreased LF power and increased HF power (Zou et al., 2018). However, these studies, based on long-term interventions (8–16 weeks), did not address changes occurring within meditation sessions. While long-term assessments are essential for understanding sustained autonomic adaptations, real-world evidence indicates that many individuals do not maintain regular meditation practice over time (Lam et al., 2023; Sullivan et al., 2023). Given the generally low persistence of meditation in the general population, understanding how individuals, beginners in particular, respond physiologically during meditation is critical. Within-session measurements can offer valuable insight into the immediate responses to different meditation types, which may shape initial user experience and influence ongoing engagement. In this context, it becomes especially important to examine how specific structural components of meditation, such as movement or verbal guidance, modulate autonomic responses during practice. Hunt et al. (2018) further explored these distinctions by dismantling MBSR components, revealing that yoga-based interventions involving movement were associated with higher resting HRV and more adaptive vagal responses to stress (Laborde et al., 2017; Christodoulou et al., 2020). This suggests that movement-based meditation promotes better stress adaptation through flexible parasympathetic responses. In contrast, mindfulness without movement resulted in more stable HRV patterns, which is interpreted as reflecting a general reduction in stress arousal rather than enhanced dynamic autonomic adaptation to environmental demands (Tang et al., 2009; Steffen et al., 2017). Dynamic autonomic adaptation refers to the capacity of the ANS to flexibly adjust physiological responses such as HRV to changing stressors, which is considered crucial for optimal stress resilience and health (Thayer et al., 2012). These distinctions underscore the need for further research on how static and kinetic meditation affect autonomic functions differently and their therapeutic implications.

Despite these insights, research on kinetic meditation remains scarce compared to that on static forms. Little is known about how these practices differentially affect ANS regulation. Given the distinct physiological responses observed, a direct comparison between static and kinetic meditation is essential to better understand their unique mechanisms and therapeutic potential. This study aimed to examine the impact of kinetic meditation on ANS regulation by comparing it with that of static meditation. By providing empirical evidence of the physiological differences between these meditation practices, this study provides valuable insights into the scientific foundation for meditation-based interventions.

## 2 Materials and methods

### 2.1 Participants

Thirty-five participants (male = 15, age: 22.03 ± 2.54 years) participated in the study. All participants were citizens of the Republic of Korea. Participants were restricted to healthy adults in their 20s who could engage in light physical activity and had no restrictions on neurophysiological measurements. Additionally, participants had to have no systematic experience in meditation practice, physical disabilities, or mental health conditions. Participants were asked whether they had (1) completed any formal course in meditation, (2) attended short-term programs such as workshops or seminars, (3) participated in one-time lectures or experiential sessions, (4) received other types of meditation instruction not mentioned above, or (5) practiced meditation regularly in daily life. Only individuals who responded “No” to all items were included in the study. Additionally, participants responded to an open-ended question regarding their reasons for not engaging in meditation prior to the study. The responses were mainly related to perceiving meditation as unnecessary, lacking time, or doubting its efficacy, indicating that participants perceived practical and cognitive barriers to meditation engagement despite having no prior experience. A within-participant experimental design was adopted to minimize individual differences related to personal meditation experience or skills. All participants completed both static and kinetic meditation sessions, with the order randomized and conducted at least 24 h apart to reduce potential carryover effects. Prior to participation, all participants provided written informed consent. This study was approved by the university's Institutional Review Board (WKIRB-202310-HR-076).

### 2.2 Measures

#### 2.2.1 Visual analog scale (VAS)

To compare the meditation experiences between static and kinetic meditation as well as between audio-guided and unguided conditions, participants were asked to respond to three questions using the Visual Analog Scale (VAS). Each participant rated their feelings on a 0–10 cm line, with endpoints labeled “not at all” and “very much.” The three items assessed were the levels of (1) focused attention (“How well were you able to stay focused without being distracted?”), (2) inner peace and calm felt (“How much inner peace and calm did you feel?”), and (3) drowsiness (“How sleepy did you feel during meditation?”) experienced during meditation. To ensure consistent interpretation among participants, particularly given their novice status, a brief explanation was provided prior to the experiment. For instance, the VAS item regarding “focused attention” was explained as: “Please consider whether your mind wandered often, or whether you were able to stay focused on your breath, bodily sensations, or other aspects of the meditation.” The concept of “drowsiness” was clarified as: “Feelings of sleepiness, such as heavy eyelids or fading awareness, including moments when you nearly fell asleep.” Participants were asked to confirm their understanding, and further clarification was provided when necessary. For analysis, the length of the mark from the left endpoint of the scale was measured using a ruler for analysis (Cline et al., 1992).

#### 2.2.2 HR and HRV

The Polar H10 (Polar Unk, Finland) was connected via Bluetooth to the EliteHRV app (EliteHRV, Asheville, USA) installed on iPhone 14 (Apple, Inc., Cupertino, CA, USA). The Polar H10 is a wireless chest strap heart rate (HR) monitor that records HR data processed by the EliteHRV app to calculate HRV metrics. Previous research has validated the reliability of measuring HRV using the Polar H10 with the EliteHRV app, with a correlation coefficient (*r*) of 0.998 or higher compared to measurements using a stationary ECG device (Im and Woo, 2024). HRV was measured during a 5-min resting-state period. Separate measurements were taken during 10-min sessions of four types of meditation: audio-guided static meditation, unguided static meditation, audio-guided kinetic meditation, and unguided kinetic meditation. The Elite HRV app recorded the data, which were automatically saved to the data log after the recording was completed. The following HR and HRV metrics were calculated and displayed: minimum HR, maximum HR, average HR, RMSSD, SDNN, natural logarithm of RMSSD (lnRMSSD), percentage of successive RR intervals that differ by more than 50 ms (PNN50), mean RR interval (the time between successive R-wave peaks), total power (TF), LF/HF ratio, LF power, and HF power. These values were coded immediately and used for analysis. The descriptions and interpretations of the HRV metrics used in this study are summarized in Table 1.

**Table 1:** Summary of HRV metrics unsed in the study.

**Domain**	**Parameter (Unit)**	**Full term**	**Description**	**Normal range (Healthy adults)**
Time-domain	RMSSD (ms)	Root mean square of successive differences	Reflects short-term HRV, highly sensitive to PNS (vagal) activity; used to assess recovery and relaxation	Mean: 29.7 (Kim and Woo, 2011); 27–58 (Shaffer and Ginsberg, 2017; Task Force of the European Society of Cardiology the North American Society of Pacing Electrophysiology, 1996)
	SDNN (ms)	Standard deviation of NN intervals	Reflects overall variability of heartbeats over a period, influenced by both SNS and PNS activity	Mean: 39.6 (Kim and Woo, 2011); 20–50 (Task Force of the European Society of Cardiology the North American Society of Pacing Electrophysiology, 1996)
	lnRMSSD	Natural logarithm of RMSSD	Used to normalize data distribution for statistical analysis	3.3–4.1 (Shaffer and Ginsberg, 2017)
	pNN50 (%)	Percentage of NN intervals >50 ms	Percentage of adjacent NN intervals differing by >50 ms; reflects vagal tone and short-term variability	3–40 (Shaffer and Ginsberg, 2017; Task Force of the European Society of Cardiology the North American Society of Pacing Electrophysiology, 1996)
	Mean RR (ms)	Mean RR interval	Average time between successive R-wave peaks; longer intervals imply lower HR and more relaxed state.	654.6–1141.4 (Smith and Smith, 1981)
Frequency-domain	TP (ms^2^)	Total power	Total variance across all frequency bands (VLF + LF + HF); reflects overall ANS activity	Mean: 1,358.9 (Kim and Woo, 2011); 1000–4000 (Shaffer and Ginsberg, 2017)
	LF (ms^2^)	Low-frequency power	Reflects both SNS and PNS activity; linked to baroreflex mechanisms and blood pressure regulation	Mean: 417.3 (Kim and Woo, 2011); 200–1000 (Shaffer and Ginsberg, 2017)
	HF (ms^2^)	High-frequency power	Linked to RSA and PNS (vagal) activity; Higher HF values reflect relaxation and rest.	Mean: 254.1 (Kim and Woo, 2011); 150–1500 (Shaffer and Ginsberg, 2017)
	LF/HF (%)	LF to HF ratio	Indicates autonomic balance; higher values suggest sympathetic dominance	Mean: 2.4 (Kim and Woo, 2011); 0.5–2.5 (Shaffer and Ginsberg, 2017; Task Force of the European Society of Cardiology the North American Society of Pacing Electrophysiology, 1996)

Values labeled as “Mean” are based on healthy Korean adults (Kim and Woo, 2011). Other ranges reflect international reference data from general adult populations. HR, heart rate; HRV, heart rate variability; ANS, autonomic nervous system; SNS, sympathetic nervous system; PNS, parasympathetic nervous system; RSA, respiratory sinus arrhythmia.

### 2.3 Procedure

The participants were instructed to abstain from caffeine, alcohol, and exercise for at least 12 h prior to each session. During the first visit, participants were provided with a detailed explanation of the study and signed an informed consent form. They then wore a Polar H10 chest strap and sat comfortably in a chair, while their baseline HRV was measured for 5 min. All sessions were conducted individually in a quiet, light-controlled environment to ensure minimal external distraction. The meditation technique used in this study was closely aligned with Focused Attention (FA) meditation, which requires sustained attention on a single object (e.g., the breath), active monitoring for distractions, and the redirection of attention when mind-wandering occurs. After the baseline measurement, participants engaged in either static or kinetic meditation, in the order randomly determined by the researcher. If static meditation was conducted first, the participants sat in a chair and performed seated breathing meditation guided by prerecorded audio for 10 min. Immediately after completing the guided meditation, they responded to VAS. Subsequently, they remained seated and performed 10 min of unguided static meditation, after which they completed the same VAS assessments. On a separate day, participants returned to the laboratory for kinetic meditation. They first engaged in a 10-min session of guided kinetic meditation involving physical movements, following instructions from prerecorded audio. Immediately after the guided session, the participants completed the same VAS assessments. This was followed by a 10-min unguided kinetic meditation session, after which they again completed the VAS assessments, marking the end of the experiment. To minimize order effects, the sequence of static and kinetic meditation sessions was randomized across participants. However, for each meditation type, guided meditation was always conducted before unguided meditation. This approach ensured that participants who were naïve to meditation could familiarize themselves with basic meditation techniques during the guided sessions before independently attempting the unguided sessions. HRV data recording began at the start of each meditation session and ended immediately upon completion, capturing HRV metrics for the entire 10-min period. The study protocol was retrospectively registered with the Clinical Research Information Service (CRIS) associated with the WHO International Clinical Trials Registry Platform (ICTRP) on 21st March 2025 (registration number: KCT0010333).

### 2.4 Statistical analysis

To examine the interaction effects of meditation type (static vs. kinetic) and guidance (guided vs. unguided), a two-way repeated-measures analysis of variance (RM-ANOVA) was conducted. Subsequently, to compare the differences in VAS scores across the four individual conditions (guided static meditation, unguided static meditation, guided kinetic meditation, and unguided kinetic meditation), a one-way RM-ANOVA was performed. To investigate differences in HRV measures (i.e., minimum HR, maximum HR, average HR, RMSSD, SDNN, lnRMSSD, PNN50, mean RR interval, TF, LF/HF ratio, LF power, and HF power) across conditions (i.e., resting-state, guided static meditation, unguided static meditation, guided kinetic meditation, and unguided kinetic meditation), a one-way RM-ANOVA was conducted, with the conditions as the independent variable and HRV metrics as the dependent variable. The effect size for all ANOVA results was calculated using an eta-squared value. Mauchly's test of sphericity was conducted for all dependent variables, and when the assumption of sphericity was not met, the degrees of freedom for ANOVA were adjusted using the Greenhouse-Geisser correction. All statistical analyses were performed using SPSS version 29 with the significance level set at 0.05.

## 3 Results

### 3.1 VAS

#### 3.1.1 Focused attention

The analysis was conducted using a two-way RM-ANOVA with a 2 (meditation type: static vs. kinetic) × 2 (guidance: guided vs. unguided) design. A significant main effect of meditation type was observed, *F*(1, 34) = 6.209, *p* = 0.018, partial η^2^ = 0.154, indicating that participants experienced greater focused attention during kinetic meditation (mean = 6.366, standard error = 0.236) than during static meditation (mean = 5.790, standard error = 0.269). A significant main effect of guidance was also found, *F*(1, 34) = 4.875, *p* = 0.034, partial η^2^ = 0.125, with participants reporting higher focused attention during audio-guided meditation (mean = 6.370, standard error = 0.220) than during unguided meditation (mean = 5.786, standard error = 0.297).

To further examine the differences across the four conditions, a one-way RM-ANOVA was conducted. The results showed a significant effect of condition on focused attention ratings, *F*(3, 102) = 4.478, *p* = 0.005, partial η^2^ = 0.116. *Post hoc* comparisons indicated that guided kinetic meditation showed significantly higher focused attention scores than unguided static meditation (*p* = 0.034) (Table 2).

**Table 2:** Mean and standard deviation of VAS scores reported across four conditions.

**VAS**	**Static meditation**		**Kinetic meditation**	
	**Guided**	**Unguided**	**Guided**	**Unguided**
Focused attention	6.01 ± 1.70	5.57 ± 1.85	6.73 ± 1.39	6.00 ± 2.07
Peace and calm	6.61 ± 1.71	5.99 ± 1.79	6.53 ± 1.58	6.19 ± 2.00
Drowsiness	3.66 ± 3.01	4.12 ± 3.12	2.31 ± 2.46	2.21 ± 2.36

#### 3.1.2 Peace and calm

For the peace and calm ratings, neither the one-way nor two-way RM-ANOVA revealed any significant differences across conditions.

#### 3.1.3 Drowsiness

The two-way RM-ANOVA revealed a significant main effect of meditation type on drowsiness, *F*(1, 34) = 14.742, *p* = 0.001, partial η^2^ = 0.288. Participants reported greater drowsiness during static meditation (mean = 3.890, standard error = 0.439) than during kinetic meditation (mean = 2.256, standard error = 0.381).

The one-way RM-ANOVA conducted on drowsiness ratings showed a significant effect of condition, *F*(3, 102) = 7.170, *p* = 0.000, partial η^2^ = 0.174. Pairwise comparisons revealed that drowsiness during unguided static meditation was significantly higher than during both guided kinetic meditation (*p* = 0.020) and unguided kinetic meditation (*p* = 0.001) (Table 2).

### 3.2 HR and HRV

**Figure 1:**
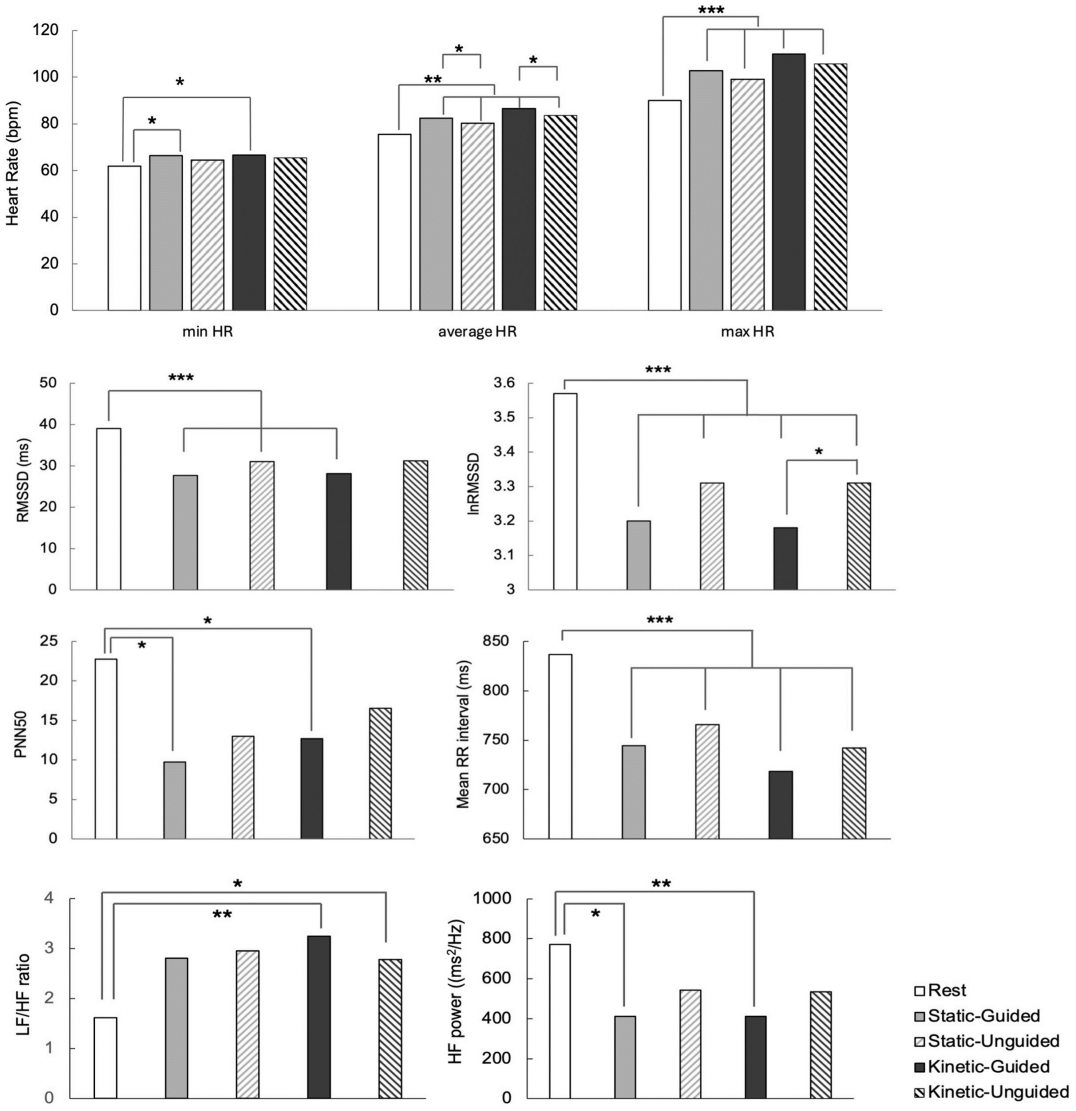
Differences in HR and HRV metrics across resting state, guide-static, unguided static, guided kinetic, and unguided kinetic meditation conditions. *, **, *** indicate statistical significance at *p* < 0.05, *p* < 0.01, and *p* < 0.001, respectively.

Differences in HR and HRV metrics across the resting state, guided static, unguided static, guided kinetic, and unguided kinetic meditation conditions are shown in Figure 1.

#### 3.2.1 Heart rate analysis

The minimum HR during guided static and guided kinetic meditation was significantly higher than that during the resting state [*F*(4, 128) = 3.031, *p* = 0.020, partial η^2^ = 0.087], while the maximum HR was significantly higher in all meditation conditions than in the resting state [*F*(4, 128) = 9.394, *p* = 0.000, partial η^2^ = 0.227]. We also observed significant differences in average HR [*F*(4, 128) = 15.431, *p* = 0.000, partial η^2^ = 0.325], where average HR during all meditation conditions was higher than that during the resting state. Additionally, the guided meditation conditions (both static and kinetic) showed a higher average HR than did their respective unguided meditation conditions.

#### 3.2.2 HRV time-domain analysis

RMSSD during guided static, unguided static, and guided kinetic meditation was significantly lower than during the resting state [*F*(4, 128) = 5.974, *p* = 0.000, partial η^2^ = 0.157]. lnRMSSD also demonstrated significant differences among conditions [*F*(4, 128) = 6.920, *p* = 0.000, partial η^2^ = 0.178], with lnRMSSD during the resting state higher than in all other conditions. Additionally, lnRMSSD was significantly higher during unguided kinetic meditation than during guided kinetic meditation. The resting-state PNN50 was higher than during guided static and guided kinetic meditation [*F*(4, 84) = 5.412, *p* = 0.001, partial η^2^ = 0.205]. Mean RR intervals showed significant differences [*F*(4, 128) = 15.250, *p* = 0.000, partial η^2^ = 0.323], with the resting state having longer mean RR intervals compared to all other conditions. However, no significant differences were observed in SDNN.

#### 3.2.3 HRV frequency-domain analysis

The LF/HF ratio was significantly higher during guided and unguided kinetic meditation conditions than during the resting state [*F*(4, 128) = 4.082, *p* = 0.004, partial η^2^ = 0.113]. HF power was significantly lower during guided static and guided kinetic meditation than during the resting state [*F*(4, 128) = 4.491, *p* = 0.002, partial η^2^ = 0.123]. However, the TF and LF powers did not differ significantly across the conditions.

## 4 Discussion

This study investigated ANS responses in novice meditators during kinetic and static meditation, along with the meditators' perceived experiences. VAS analysis revealed significant differences in participants' self-reported focused attention depending on the type of meditation and presence of guidance. Kinetic meditation led to higher levels of focused attention than did static meditation, whereas audio-guided meditation induced greater focused attention than did unguided meditation. These findings suggest that movement-based meditation and verbal guidance play crucial roles in enhancing meditators' engagement. Given that the participants in this study were beginners with no prior systematic meditation training, it appears that physical movements and audio guidance helped reduce boredom and facilitated a deeper focus. For novice practitioners, seated meditation often induces drowsiness and physical discomfort, whereas movement-based practices such as walking meditation or Hatha yoga minimize distractions from drowsiness or bodily discomfort, making continued practice easier (Kim, 2013). This claim was supported by the VAS drowsiness analysis in this study, which showed that static meditation induced significantly more drowsiness than kinetic meditation. This can be attributed to the minimal physical activity during static meditation, which may lead to greater fatigue or sleepiness.

Notably, unguided static meditation resulted in the highest level of drowsiness, suggesting that the absence of verbal cues may make it harder to maintain alertness during practice. In addition, unguided static meditation also received the lowest VAS ratings for focused attention scores. Although these two variables were not significantly correlated, the pattern of results may indicate that unguided static meditation creates conditions that are less conducive to sustained engagement, particularly for beginners. However, no significant differences were observed in peace and calm measures across meditation types or guidance conditions, indicating that these factors may have less of an influence on tranquility.

HR and HRV were analyzed across the resting state and four meditation conditions to examine the differences in autonomic responses. Both maximum and average HR were higher in all meditation conditions than at rest. The increase in HR was observed not only during kinetic meditation, which involved physical movement, but also during static meditation, suggesting that even in the absence of physical activity, meditation may impose a meaningful physiological load—particularly for beginners who may experience increased cognitive effort while sustaining attention. Specifically, the average HR was higher during guided meditation than during unguided meditation, while the minimum HR was higher during guided static and guided kinetic meditation than at rest. This pattern is consistent with previous findings that cognitive engagement—such as processing verbal instructions, integrating them cognitively, and executing the corresponding tasks—can increase SNS activity (Clark et al., 2018; Dodo and Hashimoto, 2019). Supporting this, the mean RR interval—an inverse indicator of HR—was shorter during all meditation conditions than at rest. These changes may reflects heightened physiological arousal resulting from increased attentional and cognitive demands during meditation (Hansen et al., 2003; Luft et al., 2009; Lutz et al., 2009). While meditation is often associated with relaxation, FA meditation, which was employed in this study, requires sustained attention and executive control, potentially activating brain regions involved in effortful regulation (Lutz et al., 2008; Lumma et al., 2015). From a practical standpoint, these findings suggest that guided FA-style meditation may not always induce immediate parasympathetic dominance. For novice meditators, it may initially function as a mentally demanding task that elevates HR and arousal. This is not necessarily undesirable—such activation may support cognitive engagement, especially in practices aiming to enhance attention rather than induce calm. Therefore, the physiological response profile should be interpreted in light of the practitioner's intent and experience level.

The RMSSD and lnRMSSD were significantly lower in the guided static meditation, unguided static meditation, and guided kinetic meditation conditions than in the resting state. This suggests an increase in SNS activation or a relative decrease in PNS activity during meditation. The required active engagement in meditation may have increased participants‘ mental effort, leading to heightened physiological arousal. Although unguided static meditation may appear less psychophysically demanding, novice meditators might experience elevated cognitive effort in the absence of external cues, as they attempt to self-regulate attention and maintain focus without guidance. This internal demand for attentional control may contribute to the observed reduction in HRV (Dodo and Hashimoto, 2019). In contrast, unguided kinetic meditation was the only condition in which RMSSD did not decrease relative to the resting state. This may be because movement provides a natural attentional anchor—such as proprioceptive or bodily sensations—that supports sustained engagement with reduced cognitive effort (Clark et al., 2018). Additionally, rhythmic movement may facilitate relaxation and PNS activation, buffering against the arousal seen in static conditions (Payne et al., 2015). LnRMSSD was higher for unguided kinetic meditation than for guided kinetic meditation. These findings may be attributed to a practice effect as unguided kinetic meditation was performed after the guided condition, potentially allowing participants to engage in meditation in a more familiar and relaxed state. Another possibility is that participants may have engaged in less physical movement in the unguided condition because of no instructional guidance. Alternatively, freedom from external demands in unguided kinetic meditation may facilitate greater parasympathetic activation (Mizuno et al., 2011). However, to generalize this interpretation, this study did not collect data on participants' actual movement levels in the unguided condition to compare with guided kinetic meditation, making it difficult to generalize this interpretation. Therefore, future studies should examine the extent of physical activity under different conditions.

Both guided static and kinetic meditation resulted in a significant decrease in HF power and an increase in PNN50 compared to the resting state. These results suggest a reduction in parasympathetic tone, which may occur due to stress, cognitive load, or sympathetic activation (Shaffer and Ginsberg, 2017). Given that participants reported greater focus during guided meditation based on the VAS results, the observed decrease in HF power along with an increase in PNN50 was more likely attributable to cognitive load than stress. This finding suggests that following guided instructions during meditation imposes cognitive demands that contribute to increased sympathetic activation. The LF/HF ratio in both guided and unguided kinetic meditation was significantly higher than that at rest. The LF/HF ratio is a key indicator used to assess the ANS balance. A high LF/HF ratio reflects increased sympathetic or suppressed PNS activation, which are associated with stress, tension, and the dominant action of the SNS during physical activity (Billman, 2013). This suggests that kinetic meditation involving movement activates the SNS. In particular, the elevated LF/HF ratio observed during kinetic meditation may be related to withdrawal of cardiac vagal control. As parasympathetic regulation decreases, the SNS becomes relatively dominant, which may activate mechanisms that increase HR and cardiac output (Laborde et al., 2018). This reduction in parasympathetic control could serve as a rapid physiological adaptation to meet the demands of the situation, particularly in response to physical activity (Fu and Levine, 2013; Fisher, 2014).

Taken together, the observed increase in HR and decrease in the HRV metrics during meditation in this study may be attributed to the cognitive demands placed on participants, particularly because they were meditation novices. Prior research suggests that the physiological meditation effects vary depending on the type, level of cognitive effort required, and extent of prior training (Lumma et al., 2015). Furthermore, mental effort during meditation tends to decrease with increasing expertise over time (Tang et al., 2012). Because this study did not include a comparison based on expertise, future research should examine the differences between novice and experienced meditators.

Importantly, participants in this study were not only meditation novices but also individuals who reported practical and cognitive barriers to meditation engagement, such as perceiving meditation as unnecessary, lacking time, or doubting its efficacy. In light of these characteristics, the findings provide preliminary insights for tailoring meditation interventions to populations who are both inexperienced and ambivalent toward meditation practice. Specifically, the results suggest that providing verbal guidance supports focused attention during meditation, but may concurrently increase cognitive load, as reflected by autonomic arousal markers. Kinetic meditation, which incorporates movement, appears to be particularly beneficial for beginners because it facilitates attentional engagement while reducing the drowsiness often associated with static meditation. Notably, even when performed without guidance, kinetic meditation may be easier to self-administer for novices, likely because bodily movement provides a natural attentional anchor that reduces cognitive ambiguity and supports sustained engagement. In contrast, unguided static meditation may pose greater cognitive demands for beginners, not because of task complexity per se, but due to the internal effort required to maintain attentional focus without external guidance or movement-based anchors. This increased self-regulatory load may lead to elevated drowsiness and lower attentional focus. These findings suggest that for novice meditators—especially those who initially perceive meditation as inaccessible or impractical—movement-based meditation with clear guidance may serve as an effective entry point. As practice progresses, gradually reducing external guidance while retaining a kinetic component may support the transition toward more independent meditation. Future interventions could consider adopting a stepwise approach that begins with guided kinetic meditation to build attentional capacity and reduce perceived barriers to engagement, before introducing more advanced or static forms of practice.

## 5 Limitations

This study has several limitations. First, the sample consisted of meditation novices, which may limit the generalizability of the findings to experienced meditators as autonomic responses could vary with expertise (Tang et al., 2012). In addition, although we had information about why participants had not engaged in meditation prior to the experiment, we did not assess their personal goals, expectations, or motivations when engaging in meditation during this study. This limits the ability to fully account for individual differences in anticipated meditation outcomes, which may have influenced subjective experiences or engagement. Moreover, this study did not evaluate how these experiences might have influenced their subsequent motivation to continue meditation practice or shaped their preferences for specific meditation types. Future studies should incorporate standardized assessments to more precisely capture the goals and motivations for meditation, intentions to maintain practice, and preference patterns among novice meditators. Furthermore, future research should incorporate complexity-based metrics, such as correlation dimension or multiscale entropy, to capture moment-to-moment fluctuations in heart regulation (Schubert et al., 2009; Brindle et al., 2016). Another limitation of this study is that it did not examine cardiac activity dynamics in real time within individuals (Im et al., 2024; Aro et al., 2025). Such analyses could provide deeper insights into how individuals adapt to stress during meditation, offering a more nuanced understanding of engagement and autonomic flexibility. Future research should also consider incorporating additional physiological indicators such as breath-rate variability (BRV), which may complement HRV by providing further insight into respiratory-driven components of autonomic function (Soni and Muniyandi, 2019). Since this study measured and compared the ANS responses during meditation, we were unable to identify immediate or lasting effects after meditation. Although the order of static and kinetic meditation was randomized, the sequence of guided meditation followed by unguided meditation may have influenced the results owing to potential sequence effects. While a 5-min resting HRV measurement was obtained during the first visit, physiological baselines were not reassessed before the second session (on a separate day). Given the day-to-day fluctuations in HRV, this may have affected the accuracy of between-session comparisons. Similarly, the lack of baseline VAS data constrains the accurate interpretation of session-specific changes in subjective experiences. Future studies should consider including baseline HRV and VAS assessments before each intervention session to better account for individual variability and optimize the interpretation of intervention effects. Additionally, the study did not measure or control the actual levels of physical movement during unguided kinetic meditation, which may have influenced the results. Future studies should assess physical activity levels across different conditions to better understand their impact.

## 6 Conclusions

The present study illustrates how the inclusion of movement and guidance in meditation can differentially influence ANS responses and shape novice meditators' perceptions of their meditation experiences such as focused attention, peace and calm, and drowsiness. The results indicated that focused attention was higher in kinetic meditation than in static meditation and were higher with verbal guidance. Static meditation induced more drowsiness than kinetic meditation, with unguided static meditation causing the highest level of drowsiness. Additionally, all meditation conditions increased HR and HRV markers associated with SNS activity compared to the resting state. Specifically, an increase in SNS activity during guided meditation appears to be associated with greater cognitive effort among novice meditators, leading to heightened physiological arousal. These findings suggest that beginning with movement-based meditation accompanied by clear guidance may provide a more accessible and engaging starting point for novice meditators, especially those who find meditation challenging or impractical. As practitioners become more familiar and comfortable with meditation, transitioning to static meditation could be considered, initially with guidance and eventually progressing to unguided practice. By addressing a practical gap—given that most meditation app users are beginners and that current apps often emphasize static, guided practices without sufficient empirical support—this study provides valuable evidence to inform meditation educators, app developers, and practitioners. Establishing a scientific foundation for optimizing meditation design holds promise for improving engagement and intervention efficacy across digital, face-to-face, and self-guided contexts.

## Data Availability

The raw data supporting the conclusions of this article will be made available by the authors, without undue reservation.
