# Supplementary material for: How static and kinetic meditation, with or without guidance, affect autonomic nervous system activity in novice meditators
Source: Front Psychol. 2025 Aug 5;16:1572499. doi: 10.3389/fpsyg.2025.1572499 (PMC12392108; doi:10.3389/fpsyg.2025.1572499)
Supplement: Supplementary file 1 [file Data_Sheet_1.docx]

**Meditation Protocol**

The static and kinetic meditation protocols were collaboratively developed by a certified group of four experts, including a meditation instructor, an exercise psychologist, and a yoga instructor. Both meditation types were designed to be performed while the participants were seated on a chair to ensure consistency and accessibility. Static meditation is structured as focused breathing meditation, whereas kinetic meditation involves basic yoga movements that can be performed while seated and are suitable for beginners. The audio guides for both meditation types were recorded in Korean by the same meditation instructor to maintain consistency in tone and delivery. Additionally, the scripts for the guided sessions were standardized to 430 words for both the static and kinetic meditation.

This standardized approach ensured uniformity across sessions and was tailored to suit participants with no prior experience in meditation.

**Static Meditation**

Before beginning static meditation, participants were provided with the following basic guidelines to ensure that they fully understood them:

- Sit toward the front edge of the chair with feet shoulder-width apart and back straight.
- Keep the ears, shoulders, and hip joints aligned along a straight line.
- Relax the shoulders to release tension.
- Breathe deeply through the nose and exhale slowly, allowing the breath to flow naturally.
- Avoid controlling your breath and simply observe it as it is.
- Focus inward and avoid paying excessive attention to external sounds or sensations.
- Acknowledge thoughts and emotions if they arise and gently return the focus to the breath.

Before conducting the unguided static meditation, participants were instructed to focus on their inhalation and exhalation in a relaxed manner, following the approach they practiced during the guided meditation session.

**Kinetic Meditation**

The basic guidelines for kinetic meditation are as follows:

- Ensure alignment of the ears, shoulders, and hip joints in a straight line.
- Relax the shoulders to release tension.
- Breathe deeply through the nose and exhale slowly, either through the nose or mouth, allowing breath to flow naturally.
- Avoid holding the breath during the practice.
- Perform the movements slowly and focus on the sensations in the body as one moves.
- Acknowledge emotions or thoughts if they arise and refocus on breathing, movement, and bodily sensations.

Participants were familiarized with the basic precautions before starting kinetic meditation. As they had to rely solely on the audio guide to perform the required movements, the three poses included in the meditation (mountain, tree, and warrior poses) and their variations were shown using pictures. The researcher also demonstrated each movement to ensure participants were adequately familiar with it. Details of the movements included in kinetic meditation are provided in Supplementary Table 1. These movements were designed for simplicity and accessibility even for beginners, while encouraging a mindful focus on body alignment and breathing throughout the practice. The kinetic meditation session lasted 10 min, incorporating breathing and alignment during opening, closing, and transitions between movements.

During the unguided kinetic meditation, the participants were allowed to perform any movements, whether included in the previous audio-guided kinetic meditation session or freely chosen based on their preference, as long as they remained seated in the chair. They were encouraged to move slowly, coordinate their movements with their breath, and focus mindfully on their bodily sensations as well as any emotions or thoughts that arose during the session.

**Supplementary Table 1**. Movement and Time Structure of Guided Kinetic Meditation

| Movement | Time |
| --- | --- |
| - (Mountain Pose) spread and raise the arms up parallel above the head, then lower them back to the sides. | Slowly repeat for 1 min |
| - Gently open and close the fists. | Slowly repeat for 30 s |
| - Rotate the wrists clockwise and counterclockwise. | Slowly repeat for 30 s |
| - Alternately straighten one leg at a time and lift the foot off the ground. | Slowly repeat for 1 min |
| - (Tree Pose) Lift one foot and place it on the opposite thigh, bringing the hands together in front of the chest. | Hold for 30 s |
| - Extend the arms straight, relax the shoulders, and hold the pose. | Hold for 20 s |
| - Switch legs and repeat the same pose. | Hold for 30 + 20 s |
| - (Warrior Pose) Stretch the left leg out to the side, keeping the hands on the hips. | Hold for 20 s |
| - Extend the arms out to the sides and gaze beyond the right hand. | Hold for 30 s |
| - Switch sides and repeat the same pose. | Hold for 20 + 30 s |
